# Tracheotomy in Diphtheritic Laryngitis

**Published:** 1881-12

**Authors:** J. W. Chambers

**Affiliations:** Baltimore, Md.; Demonstrator of Anatomy, College of Physicians and Surgeons, Baltimore, Maryland


ATLANTA

MEDICAL REGISTER.

VOL. I.] DECEMBER, 1881. [No. 3

### Original.

### TRACHEOTOMY IN DIPHTHERITIC LARYNGITIS.

BY J. W. CHAMBERS, M.D., BALTIMORE, MD.
Demonstrator of Anatomy, College of Physicians and Surgeons, Baltimore, Maryland.

*Read before the Baltimore Medical and Surgical Society Oct.* 12, 1881.

Under this heading I shall simply relate the cases that have come under my observation, fully, but concisely and uncolored, with a few suggested remarks. They will exhibit the ordinary types of membranous croup as it has occured in our city at different times in the last year and a half. I shall assume without argument that there is no difference between what is generally called true croup and diphtheritic laryngitis, therefore in the below reported cases I have made no effort to make the distinctive diagnosis.

Case I.—S. M., female, white, age fourteen months, previously healthy, had been sick for three days with diphtheritic pharyngitis. At the beginning of the fourth day she began to grow hoarse, coughed croupy; during the following night she had several attacks of dyspnœa; emetics and the common remedies that are used in such cases were then freely given, but gave negative results. I saw her on the fifth day with the attending physician, Dr. Coyner. She, at that time, labored under stridulous respirations.

There was supra-clavicular and epigastric depression at every inspiration, pulse 140, respirations 60, temperature 104. Lungs on percussion gave no evidence of any complication; auscultation impractible on account of the laryngeal sounds. Tracheotomy under an anæsthetic was at once performed—Drs. S. F. Coyner and J. B. Boyle assisting.

The relief was complete and everything went on favorably for 36 hours. Respiration and pulse became less frequent and the temperature fell to 100; she took milk and stimulants freely.

The child was doing so well that all apprehension was fast passing away. The mother, who was a very nervous and hysterical lady, was left to attend the child. Suddenly it gave a cough, rose up in bed, gasping for breath and pulling at the neck. The mother became frightened and ran out of the room for help; when she returned the child was dead—all from the want of an experienced, competent and reliable nurse. On examination of the tube after death it was completely occluded with a piece of membrane.

Case II.—M. J., two years and a half old, had been sick with sore throat for six days, which had been growing worse when Dr. Taylor was called to see a fully developed case of croup. I saw the case, with Dr. Taylor, on the eighth day; the fauces and the whole throat were covered with a thick, dirty-white looking membrane. Respiration 40, and labored; pulse 160, temperature 103°. At every effort of respiration the sternum would almost meet the vertebral column. Face pale, cold and perspiring; lips and fingers blue. Drs. Parker and Taylor assisted me in performing tracheotomy. The operation was simple and bloodless, and gave complete relief to the urgent symptoms—dyspnœa, etc. Child died quietly thirty-six hours after the operation.

Case III.—C. H., male, æt. 4 years, had been sick two days when Dr. Taylor was called to see him. He found him with pharyngeal diphtheria and a croupy cough. In the next few hours he developed symptoms of laryngeal obstruction, which had increased to such a degree by the following day as to warrant tracheotomy. The tonsils and pharynx were congested and partially covered with a diphtheritic deposit. Respiration 40, pulse 140, weak and feeble; temperature 102°. With the assistance of Drs. Taylor and Free, the operation was performed, which was attended by no accidents. Complete relief to symptoms occurred, and the boy went off into a refreshing sleep. Nothing of special interest occurred until the eighth day, when it was deemed safe to remove the tube, after which the wound closed without special treatment. Three weeks after the operation the boy was playing around, well.

Case IV.—J. D., male, æt. three and one-half years, previously healthy, complained of sore throat for some days before the calling in of Prof. Lynch, who, after examining the throat, with the other symptoms, pronounced the case one of true membranous croup. Emetics and the other usual remedies were resorted to, but with no avail. I saw the patient on the fourth day. He was a strong, robust looking boy, suffering from intense dyspnœa, quite unable to speak. Respirations, 50 per minute; cartilages of the ribs and sternum drawn in at every effort to breathe; pulse, 160; temperature, 103°; lips of a dark livid color; cough constant and brassy. Tracheotomy was performed with complete arrest of all the most formidable symptoms; temperature fell to 100°; pulse, 110; taking nourishment freely and frequently; everything going on nicely until the fourth day after operation, when the temperature ran up to 104°; respirations became frequent and panting; pulse, 180; irritating cough; lungs gave indications of a diffuse capillary bronchitis, from which he died on the sixth day after the operation.

Case V.—M. G., female, æt. three years, complained with sore throat on Wednesday. Dr. Fishburne was called in on Thursday, and pronounced it a case of diphtheria. Case did very well until Saturday morning; he noticed the cough somewhat brassy, and respiration slightly labored. These symptoms grew rapidly worse, until at 8 o'clock, in consultation with Dr. Clagett, tracheotomy was advised, which I did with the assistance of Dr. Fishburne, Messrs. Shackelford and Berst, medical students. No accident occurred during its performance, and the relief was complete. The following day the respirations again became hurried and labored, and all of the dreaded symptoms returned. The patient died fifty-six hours after the operation, from the extension of the disease down into the bronchial tubes.

Case VI.—Male, æt. fourteen months, strong and healthy; was called to him for sore throat. Diagnosticated diphtheria; gave him iron and quinia internally, with acid carbol., alcohol and glycerine, as a local application. Membrane had disappeared; patient was doing well, in fact was thought out of danger, when on Monday it was noticed that he coughed somewhat croupy. On examining his throat some patches of membrane were noticed on the posterior fauces. Laryngeal symptoms grew more marked; cough still more brassy; respirations more labored, until with every breath there was depression of sternum and supra-clavicular regions. Tracheotomy was at once performed, with the assistance of Dr. Parker and Mr. Van Note, medical student. The neck was very thick and short, trachea deep; no hemorrhage or other accidents. The child rallied well from under the anæsthetic, breathed easily and noiselessly, slept for some hours free from all the untoward symptoms. Twelve hours after operation respirations again became labored and noisy, and the patient died from the extension of the membrane down the trachea, eighteen hours after the operation.

Case VII.—W. P., male, æt. three years, had been attended some six days for diphtheritic pharyngitis, and was considered out of danger by the attending physician, Dr. Russell. The membrane had nearly all disappeared from the fauces; temperature normal; pulse, 100. Towards the evening of the seventh day he became restless, feverish and thirsty; developed hoarseness; in the course of the next few hours had one or two attacks of dyspnœa. The doctor was summoned, and he pronounced it diphtheritic croup, and gave the family but little hope of a recovery. I saw him on the evening of the following day. He had some little swelling of the glands of the neck; there was a dark, grayish, dirty-looking membrane on the soft palate and pharynx; none noticed in the nasal passages.

Respirations 40, rapid and labored, and accompanied by the usual symptoms of laryngeal stenosis; pulse 150, weak and feeble; temperature, 102°. Lungs were tympanitic, and on aucultation the vesicular murmur could be heard over the area of the entire chest.

With the assistance of Dr. Russell and Mr. Van Note, medical student, I performed tracheotomy at once. The trachea was reached without much difficulty. No accidents; hemorrhage slight and was readily arrested after the introduction of the tube, which was accompanied by the welling up of purulent matter and several pieces of membrane. After this was accomplished the child breathed quietly and noiselessly, dropping off into a quiet, easy sleep. The following morning, eight hours after the operation, there was still some swelling of the neck, and membrane noticed in the fauces, but otherwise the symptoms were favorable. Temperature, 100.5°; pulse, 120; respirations, 30, and easy. His condition continued favorable, with now and then a slight change in the respirations, temperature and pulse, due to some accidental cause. On the ninth day after the operation the tube was removed, and the boy made a complete and rapid recovery.

In all of the above cases the constitutional treatment, which was kept up with as much regularity as though tracheotomy had not been done, consisted of quinia, tincture of iron, and brandy *ad libitum*, with inhalations of lime water, etc.

At the present day we would not be justified in narrowing the operation for the division of the trachea to any single method. From the statistics of the operation I gather that probably the high operation is the one most frequently performed. This is explained by the easier performance of the operation at this point since the anatomical guides are more prominent. One of the objections, however, is the narrowness of the trachea at this point, and the pressure of the tube may consequently produce gangrene of the upper rings of the trachea and cricoid cartilage. The operation below the gland has the advantage that in childhood that portion of the wind-pipe is largest in caliber and is more apt to be below the obstuctive exudation. But it also has the disadvantages of being near the large and important vessels and altogether impracticable in fat, short necked children.

In three of my cases I have gone through the isthmus of the thyroid gland and have been quite satisfied with the results, in fact I should consider it the point of election, of course to be modified by the peculiarities of each case. There is not that danger if cut through the median line as was once thought, since by experiments we are unable to inject the isthmus from either side, the vessels of each side being entirely separated as in the raphe of the perineum. As it has all of the advantages of the high or low operation and none of the dangers of either, I shall in the future adhere to the median operation when not contra-indicated.

Relative to the tubes; in my experience all that are in common use have been as a rule too small in caliber and not of sufficient curve. The ideal tube should be as large as any given trachea will comfortably admit, with a curve about equal to Thompson's catheter so that the friction of the tube against the wall of the trachea shall be brought to a minimum.

As to when the operation should be done, it might be simply stated, in the first and second stages, but it is strictly speaking, a clinical question and therefore can only be answered by the judgment of the practitioner.

The varied and frequent objections to tracheotomy as a remedial agent in diptheritic laryngitis in the face of modern surgery and statistics are but the low and distant mutterings of the passing storm, and the place that it now holds in the well recognized life-saving procedures which are resorted to by all earnest practitioners of medicine and surgery make it not only justifiable but imperative; and in many cases the only expedient that can offer the least hope of saving life. In all such cases he who neglects or fails to urge the operation is remiss in his duty to his patient as well as to his profession. Governed by these considerations I feel it incumbent and shall always advise and urge tracheotomy when the gravity of the circumstances seem to me to demand it.

